# Cast versus removable orthosis for the management of stable type B ankle fractures: a systematic review and meta-analysis

**DOI:** 10.1007/s00068-022-02169-6

**Published:** 2022-11-16

**Authors:** Jelle Friso Spierings, Thomas Marcus Paulus Nijdam, Lizz van der Heijden, Henk Jan Schuijt, Marike Cornelia Kokke, Detlef van der Velde, Diederik Pieter Johan Smeeing

**Affiliations:** 1 Department of Trauma Surgery, St. Antonius Hospital, Soestwetering 1, 3543 AZ Utrecht, The Netherlands; 2 Department of Orthopedic Surgery, Leiden University Medical Center, Albinusdreef 2, 2333 ZA Leiden, The Netherlands

**Keywords:** Weber B, Lauge-Hansen, Cast, Ankle fracture, Orthosis, Immobilization

## Abstract

**Purpose:**

There is currently no consensus on nonoperative management in adult patients after a stable type B ankle fracture. The aim of this review is to compare a removable orthosis versus a cast regarding safety and functional outcome in the NOM of stable type B ankle fractures.

**Methods:**

A systematic review and meta-analysis were performed using randomized clinical trials and observational studies. The methodological quality of the included studies was assessed with the methodological index for non-randomized studies instrument. Nonoperative management was compared using the number of complications and functional outcome measured using the Olerud and Molander Score (OMAS) or the American Academy of Orthopaedic Surgeons Ankle Score.

**Results:**

Five studies were included. Two were randomized clinical trials, and three were observational studies, including a total of 516 patients. A meta-analysis showed statistically significant higher odds of developing complications in the cast group [odds ratio (OR), 4.67 (95% confidence interval (CI) 1.52–14.35)].

Functional outcome in OMAS did not vary significantly at 6 weeks, mean difference (MD) − 6.64 (95% CI − 13.72 to + 0.45), and at 12 weeks, MD − 6.91 (95% CI − 18.73 to + 4.91). The mean difference of functional outcome in OMAS at 26 weeks or longer was significantly better in the removable orthosis group; MD − 2.63 (95% CI − 5.01 to − 0.25).

**Conclusion:**

Results of this systematic review and meta-analysis show that a removable orthosis is a safe alternative type of NOM, as complication numbers are significantly lower in the orthosis group. In addition, no statistically significant differences were found in terms of functional outcome between a removable orthosis and a cast at 6 and 12 weeks. The 6-week and the 26-week OMAS results show that in patients with stable type B ankle fractures, a removable orthosis is non-inferior to a cast in terms of functional outcome.

## Introduction

The incidence of ankle fractures is between 160–187 fractures per 100,000 person-years and is rising [1–3]. Ankle fractures are the most common lower extremity fracture, and two-thirds of all ankle fractures are fractures of the distal fibula at the level of the tibiofibular syndesmosis [4, 5]. These injuries are caused by supination-external rotation trauma and are considered stable if there is no additional medial or posterior injury [6, 7]. Stability assessment of these fractures is crucial, as it dictates further treatment strategy [8, 9]. The most commonly used methods for stability assessment are Mortise radiographs with additional criteria, external stress rotation radiographs, and gravity stress radiographs [10]. Stable fractures are classified as type 44-B1 (type B) ankle fractures by the AO Foundation/Orthopedic Trauma Association (AO/OTA), or as Lauge-Hansen supination-external rotation stage II, or as stable, isolated Weber B ankle fractures [11].

Type B fractures have a major impact on affected individuals, families, social life, and societal costs due to (occupational) disability and loss of independence for prolonged periods [12]. Traditionally, nonoperative management (NOM) of these fractures involves a below-the-knee immobilization for 6 weeks, consisting of 4 weeks of non-weight-bearing followed by 2 weeks of weight-bearing, or 6 weeks of non-weight-bearing. This type of NOM facilitates bone union but can result in joint stiffness, muscle wasting, or deep venous thrombosis (DVT) [13–15].

Removable orthoses have proven their value in the recovery process of other fractures of the long bones, and are increasingly suggested as an alternative to a cast for stable type B fractures [16, 17]. Orthoses can be taken off, allowing early (weight-bearing) mobilization, potentially preventing the consequences of rigid immobilization and accelerating recovery [16, 18].

Both types of NOM have benefits and drawbacks, but no consensus on NOM for stable type B ankle fractures has been reached. The aim of this review was to compare a removable orthosis versus a cast regarding safety and functional outcome in the NOM of stable type B ankle fractures.

## Methods

### Study design

No ethical committee approval was necessary for this study. No published protocol for this review exists. This systematic review and meta-analysis was performed according to the Preferred Reporting Items for Systematic Reviews and Meta-analysis Statement (PRISMA) [19].

### Search strategy and selection of studies

A systematic PubMed, Embase and Cochrane literature search was conducted on the 15th of July 2022. All relevant synonyms for two search terms, ‘ankle fracture’ and ‘orthosis’ were included in the search syntax (Appendix 1). Studies were included if: (1) NOM with a cast versus removable orthosis after stable type B ankle fractures were compared, (2) included patients were skeletally mature (16 years or older), (3) complications (e.g. DVT, stiffness) and functional outcome measured in Patient Reported Outcome Measures (PROMs) up to 52 weeks were described, (4) available in Dutch, English, or German, (5) original data was reported (no reviews, editorial letters, or expert opinions). Rayyan was used for data management and selection of the studies [20]. After the removal of duplicates, titles and abstracts were screened for relevance. If full-texts were not obtained via PubMed or the journal of publication and the author of the article had not been reached, the additional databanks from Google Scholar and Research Gate were searched. Obtained full-texts of eligible studies were read before final inclusion. Screening for relevance by title, abstract and full text before final inclusion has been performed by two independent researchers (JS and TN). Reference checking was performed to search for additional relevant studies.

### Assessment of methodological quality

Two independent researchers (JS and TN) assessed the methodological quality according to the Methodological Index for Non-Randomized Studies (MINORS) for all full-texts of included studies [21]. The MINORS score for comparative studies ranges from 0 to 24, with a higher score representing a better methodological quality.

For this review, a score of less than 14 was considered poor quality, 15–19 moderate quality, and 20–24 for good quality for comparative studies [22]. Other quality-assessment tools focus on a specific study design, while the MINORS is externally validated on RCTs making it a suitable instrument for meta-analyses of different study designs [21]. This index is validated to assess the quality of non-randomized and randomized studies.

Disagreements were resolved by discussion with a third reviewer (DS) until consensus was reached.

### Data extraction

The following paper-related data were extracted: first author, year of publication, country in which the study was conducted, and study design [e.g. cohort study, randomized clinical trial (RCT)]. Extracted patient-related data were: included number of patients, age in years, sex of included patients, number of patients treated with a removable orthosis, type of orthosis (e.g. walker, air-stirrup brace), number of patients treated with a cast, duration of NOM in weeks (e.g. immobilization in cast in weeks). Extracted diagnostic criteria to assess fracture stability were extracted: type of diagnostics (radiograph, CT, ultrasound), classification system (Lauge-Hansen, Weber), sub-classification (Supination External Rotation type II, Weber B), additional radiological criteria [Superior Clear Space (SCS) in millimetre (mm)], Medial Clear Space (MCS) in mm, MCS and SCS ratio, fibular dislocation in mm, MCS under external rotation stress in mm, additional clinical diagnostic criteria (e.g. medial side tenderness), time to performance diagnostics in days (e.g. at primary visit, or 10–14 days after trauma). The following weight-bearing related data were extracted: weight-bearing permission by a medical specialist (yes/no), percentage of allowed weight-bearing in the affected limb (0–100), time to start weight-bearing after injury in days, reported number and type of complications, including Confidence Intervals (CI) or, if CIs are unavailable *p* values, used scale and timing of reported functional outcomes (e.g. PROMs, including CIs and *p* values). Contact with the corresponding author was attempted for missing data. Additional data was successfully obtained from van den Berg et al. [23].

### Outcome measures

The safety of NOM during posttraumatic care management was evaluated using the reported number of complications up to one year after injury. Complications of NOM consisted of persistent joint stiffness, pressure injuries caused by orthosis, or casts (epidermal excoriation, dermal excoriation), persistent pain [Visual Analogue Scale (VAS) lower than 3], persistent perceived instability at final follow-up, failure of immobilization material (e.g. breakage), or clinically diagnosed DVT. Complications requiring surgical intervention were delayed or non-union, or secondary displacement. Delayed union was defined as incomplete radiographic healing after 6 months. A patient was considered to have a non-union if incomplete radiographic healing remained after 12 months.

Functional outcomes of studies were evaluated, obtained and included two patient-reported outcome measures (PROMs): the American Academy of Orthopaedic Surgeons Ankle Score (AAOS) and Olerud Molander Score (OMAS) [24, 25].

### Data analysis

Data management, statistical analyses, and graphical representation were performed using Review Manager software (RevMan v 5.4.1) provided by the Cochrane Collaboration [26]. Study results were reported by absolute numbers with percentages. Outcomes reported by two or more studies were pooled in a meta-analysis. To perform a meta-analysis, missing means or SDs that were not reported in an article and which could not be retrieved after the attempt to contact the corresponding author were estimated if possible using the available information. If the range was available for outcome variables, the SD was estimated as range divided by 4 [27]. The SD was estimated from the standard error (SE) using the formula SE = SD/√*n*, where *n* is the sample size. The SE was estimated from the 95% CI by dividing the width of the CI by 2 × 1.96 = 3.92 [27]. Data were converted into the same units if needed.

The assessment of statistical heterogeneity was performed by estimating statistical measures of heterogeneity: Cochran *Q* (Chi-square test), *I*^2^, and *τ*^2^ (tau-square). The random-effects model was used for meta-analyses. The overall-effect *Z* test was used to determine significance. A two-sided *p *value of 0.05 or less was considered statistically significant. The inverse variance statistical method was used to construct a 95% CI. A pooled odds ratio (OR) was estimated for dichotomous outcomes. The Mantel–Haenszel statistical method was used to construct a 95% CI [28]. A mean clinically important difference (MCID) was set at 4.4 points on the OMAS scale as suggested by Nilsson et al. [29]. The data of OMAS are presented as mean between-group difference (with 95% confidence interval). A negative difference means that the participants in the removable orthosis group reported higher OMAS for that time point. For the secondary outcome, functional outcome, 12 weeks follow-up was considered the most appropriate timing for measuring OMAS. Other time points were OMAS at 6 weeks or the closest shorter timing of follow-up (e.g. 4 weeks), and OMAS at 26 weeks or the closest timing of later time points (e.g. 52 weeks).

## Results

### Search

**Fig. 1:**
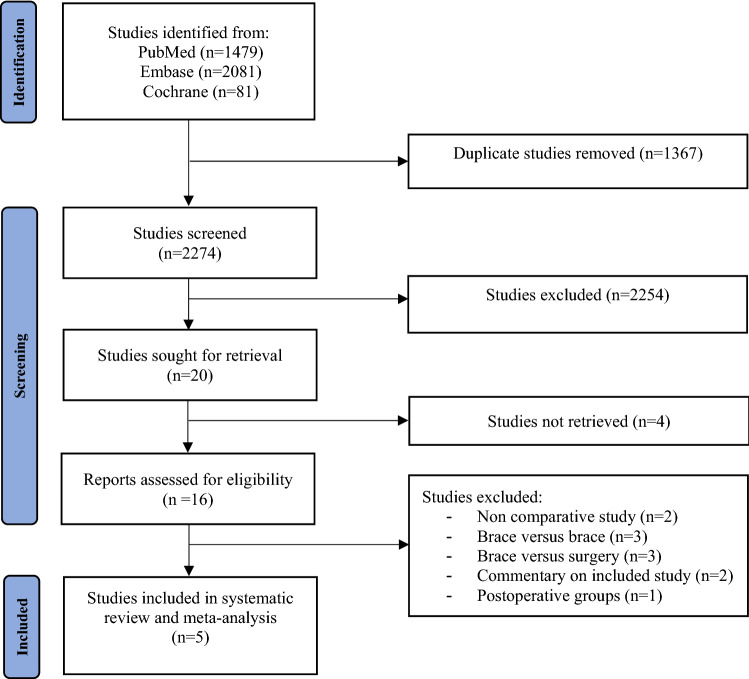
PRISMA flow diagram of search process in a systematic review and meta-analysis of stable type B ankle fractures comparing removable orthosis and casting

In total, 1479 (PubMed), 2081 (Embase), and 81 (Cochrane) articles were identified. After removing duplicates, 2274 articles were screened for title and abstract. A total of 20 full text of studies were screened for eligibility, of which five were included [23, 30–33]. Of all included studies, two were RCTs [23, 32], and three were observational studies [30, 31, 33]. Of these observational studies, two were prospective studies [31, 33], and one was a retrospective study [30]. The study selection is illustrated in Fig. 1 using the PRISMA flow diagram.

### Baseline characteristics

A total of 516 patients (43% male) with a mean age of 44 years (ranging from 16 to 92 years) were included. Studies did not apply different inclusion or exclusion criteria for the NOM-groups in terms of age, comorbidities and time of presentation after trauma. Sample size ranged from 37 to 247 patients. A total of 303 patients were immobilized with a cast versus 213 patients treated with an orthosis. All studies reported lower rates of male participants, in particular the study of Port et al. (29%) [31]. Detailed study characteristics are described in Table 1. The type of removable orthosis varied from elastic support bandages to long walking boots (Table 2). Two studies used the Lauge-Hansen classification system to describe fracture types [31, 33], and three studies used the Weber classification with additional criteria [23, 30, 32]. Definition of clinical or radiological stability was described in four out of five included studies and varied throughout all studies (Table 3) [23, 30–32].

**Table 1:** Study characteristics of the included studies comparing removable orthosis and casting in patients with a stable type B ankle fracture

First author	Year	Country	Study design	Number of patients	Fracture classification	Mean age in years (range)	% of patients male
Abdelaal [30]	2021	United Kingdom	Retrospective cohort study	123	Weber	52 (18–92)	43
Kortekangas^c^ [32]	2019	Finland	Multicentre RCT^a^	247	Weber	45 (16–85)	51
Stuart [33]	1989	United Kingdom	Prospective cohort study	37	Lauge-Hansen	N/A^b^	N/A^b^
van den Berg [23]	2018	Netherlands	RCT^a^	44	Weber	43 (18–74)	55
Port [31]	1996	England	Prospective cohort study	65	Lauge-Hansen	48 (18–87)	29

^a^Randomized clinical trial

^b^Not available

^c^The study of Kortekangas et al. described three groups. Orthosis during three weeks has been compared with a cast of 3 weeks

**Table 2:** Immobilization type and distribution in studies included in a systematic review of stable type B ankle fractures comparing removable orthosis and cast as nonoperative management

Study	Patients treated with removable orthosis; *n* (%)	Type of removable orthosis	Patients treated with cast; *n* (%)	Duration of nonoperative management in weeks
Abdelaal [30]	61 (49.6)	Long walking boot	62 (50.4)	6 weeks
Kortekangas [32]	80 (32.3)	Dynacast-orthoglass	3 weeks: 83 (33.6)^a^ 6 weeks: 84 (34)	Orthosis 3 weeks Cast 3 or 6 weeks^a^
Stuart [33]	19 (51.4)	Air stirrup	18 (48.6)	4 weeks
van den Berg [23]	23 (52.3)	Bauerfeind malleoloc	21 (47.7)	6 weeks
Port [31]	30 (46.2)	Elastic support bandage	35 (53.8)	4 weeks

^a^The study of Kortekangas et al. described three groups. Orthosis during three weeks has been compared with cast of three weeks

**Table 3:** Definition of stability used in a systematic review of stable type B ankle fractures comparing removable orthosis and cast as nonoperative management

Study	Stability test	Criteria of stability	Timing of testing (in days)	Weight-bearing^b^
Abdelaal [30]	Weight-bearing radiograph	MCS^a^ < SCS^d^	Secondary visit (7–14)	Yes, after stability test
Kortekangas [32]	External rotation stress test under fluoroscopy	MCS^a^ < 5 mm under external rotation stress	Primary visit	Yes, immediate
Stuart [33]	Anterior posterior Radiograph	No medial side tenderness, unclear radiographic criteria	Primary visit	Yes, after 48 h
van den Berg [23]	Mortise and lateral Radiograph	MCS^a^ < 4 mm and (MCS^a^ < SCS^d^ + 1 mm) Dislocation < 2 mm on Mortise + lateral x-rays	Primary visit	No, non-weight-bearing splint
	Mortise and lateral Radiograph	MCS^a^ < 4 mm and (MCS^a^ < SCS^d^ + 1 mm) Dislocation < 2 mm on Mortise view + lateral x-rays	Secondary visit (7)	Yes, after stability test
Port [31]	Radiograph	No medial side tenderness, unclear radiographic criteria	N/A^c^	Yes, immediate

^a^Medial clear space

^b^Superior clear space

^c^Not available

### Assessment of methodological quality

Table 4 shows the distribution of the study quality across the studies. Three studies were of poor quality [30, 31, 33] and both RCTs were of good quality [23, 32].

**Table 4:** Risk of bias appraisal following MINORS criteria of studies included in a systematic review of stable type B ankle fractures comparing removable orthosis and cast as nonoperative management

	A stated aim of the study	Inclusion of consecutive patients	Prospective collection of data	Endpoint appropriate to the study aim	Unbiased evaluation of endpoint	Follow-up period appropriate to the major endpoint	Loss to follow-up < 5%	Prospective sample size calculation	Gold standard for control group	Contemporary groups	Baseline equivalence	Statistical analysis for study design	Total score
Abdelaal [30]	1	2	0	2	0	0	0	0	0	2	1	1	10
Kortekangas [32]	2	1	2	2	1	2	0	2	2	2	2	2	20
Stuart [33]	1	1	2	1	1	1	0	0	0	2	0	0	9
van den Berg [23]	2	1	2	2	2	2	0	2	2	2	2	2	21
Port [31]	1	1	2	1	1	2	0	0	1	2	1	0	12

0 indicating that it was not reported in the article evaluated, 1 indicating that it was reported but inadequately, and 2 indicating that it was reported adequately

### Safety

**Fig. 2:**
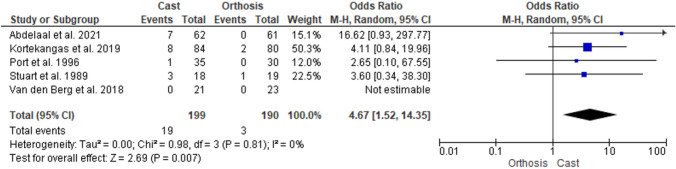
Complications in a systematic review of type B ankle fractures comparing removable orthosis and a cast. *The 3-week cast group of Kortekangas et al. was compared with the 3-week orthosis group. The 6-week cast has not been used during this analysis

Complications were statistically significantly higher in the cast group (OR 4.67, 95% CI 1.52–14.35) (Fig. 2). DVTs occurred in 11 patients in the cast group versus none in the removable orthosis group. The cast group in van den Berg et al., was the only treatment group to receive thromboprophylaxis [23]. Other reported cast related complications were nerve compression (*n* = 2), non-union (*n* = 2), secondary displacement (*n* = 3), cast related pain (*n* = 2), delayed union (*n* = 2), and breakage of immobilization (*n* = 1). Only Abdelaal et al. reported failure of NOM [30]. Three patients in the cast group developed secondary displacement, requiring open reduction and internal fixation (Table 5) [30].

**Table 5:** Complications and functional outcomes in a systematic review of stable type B ankle fractures comparing removable orthosis and cast as a nonoperative treatment regimen

Study	Removable orthosis			Cast		
	Type of complications; *n* (%)	PROMs (timing in weeks)	Mean score (range or SD)	Type of complications; *n* (%)	PROMs (timing in weeks)	Mean score (*n*, range or SD)
Abdelaal [30]	0 (0)	OMAS^b^ (≥ 26)	92.2 (75–100)	DVT^c^, 1 (2) Delayed union, 1 (2) Displacement 3 (5)	OMAS^b^ (≥ 26)	90 (45, 78–100)
Kortekangas [32]	Nerve compression 2 (1)	OMAS^b^ (6) OMAS^b^ (12) OMAS^b^ (52)	55 (± 20)^a^ 70 (± 21)^a^ 89.8 (± 18.3)^a^	DVT^c^, 3 (4) Non-union, 2 (2) Nerve compression, 1 (1)	OMAS^b^ (6) OMAS^b^ (12) OMAS^b^ (52)^e^	54 (± 18)^a^ 73 (± 19)^a^ 91.7 (± 12.9)^a^
				DVT^c^, 5 (6) Cast pain, 2 (2) Nerve compression, 1 (1)	OMAS^b^ (52)^f^	87.6 (± 18.4)^a^
Stuart [33]	Superficial ulcer 1 (4)	N/A^d^	N/A^d^	DVT^c^, 2 (11) Breakage of cast 1 (5)	N/A^d^	N/A^d^
van den Berg [23]	0 (0)	OMAS^b^ (6) OMAS^b^ (12) OMAS^b^ (26) AAOS (6)	61.3 (± 22.3)^a^ 78.1 (± 22.5)^a^ 90.4 (± 17.1)^a^ 95 (N/A)^d^	0 (0)	OMAS^b^ (6) OMAS^b^ (12) OMAS^b^ (26) AAOS^g^ (6)	51.8 (± 22.3) 64.3 (± 24.9) 79.6 (± 23.1) 91.6 (N/A)^d^
Port [31]	0 (0)	OMAS^b^ (26)	93 (± 1.2)^a^	Delayed union 1 (3)	OMAS^b^ (26)	89 (± 3.8)^a^

^a^Standard deviation, no range was described

^b^Olerud and Molander Score

^c^Deep venous thrombosis

^d^Not available

^e^Three-week immobilization

^f^Six-week immobilization

^g^American Association of Orthopaedic Surgery

### Functional outcome

**Fig. 3:**
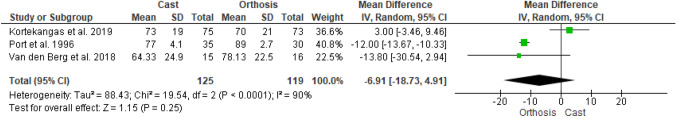
The mean difference (SD) on functional outcomes measured in OMAS after type B ankle fractures comparing removable orthosis and a cast at 12 weeks. The data is presented as mean between-group difference (with 95% confidence interval) at 12 weeks. A negative difference means that the participants in the removable orthosis group reported higher OMAS at the 12-week point than the cast group. *The 3-week group of Kortekangas et al. was compared with the 3-week orthosis group. The 6-week cast group has not been used during this analysis

All included studies reported functional outcomes. Functional outcome was assessed through PROMs in four studies. No statistically significant differences in OMAS were found between a cast or removable orthosis at 6 weeks and 12 weeks, with a mean difference (MD) of − 6.64 (95% CI − 13.72 to + 0.45) at 6 weeks[23, 31, 32], and -− 6.91 (95% CI − 18.73 to + 4.91) at 12 weeks, both in favour of the removable orthosis [23, 31, 32]. A more detailed description of the MD at 12 weeks can be found in Fig. 3. A statistically significantly difference in OMAS was found between a cast or removable orthosis at 26 weeks in favour of the removable orthosis, with an MD of − 2.63 (95% CI − 5.01 to − 0.25) [23, 30–32]. The 95% CI of the 6-week and the 26-week OMAS results do not overlap the MCID of 4.4 points, showing that in patients with stable type B ankle fractures, a removable orthosis is non-inferior to a cast in terms of functional outcome. No statistically significant differences were found in the AAOS used in one study, with a mean score of 92 in the cast group, and 95 for the removable orthosis group (*p* = 0.39) [23]. Timing of the PROMs as an outcome measure varied from 6 to 52 weeks after injury.

Stuart et al. described functional outcome as symptom-free after 3 months in range of motion, pain, and level of activity [33]. A higher percentage of patients in the orthosis group (*n* = 16, 84%) was symptom-free compared to the cast group (*n* = 12, 66%) [33].

## Discussion

Results of this systematic review and meta-analysis show that a removable orthosis is a safe alternative type of NOM, as complication numbers are significantly lower in the orthosis group. In addition, a removable orthosis shows no statistically significantly differences compared to a cast in terms of functional outcomes in patients with stable type B ankle fractures at 6 or 12 weeks follow-up. The 6-week and the 26-week OMAS results show that in patients with stable type B ankle fractures, a removable orthosis is non-inferior to a cast in terms of functional outcome.

### Comparison with other studies

This review found fewer complications in the orthosis group compared to the cast group and similarly high scores of functional outcome in both NOM-groups, partly in keeping with previous literature.

Several other studies, which were not included in this review, described complications after management with a removable orthosis or cast after an ankle fracture. These studies had several important differences in study design [34–37]. They were either non-comparative, used cast or orthosis as post-operative management, or included more types of ankle fractures [34–37]. Studies comparing a removable orthosis and cast as post-operative management have described similar high scores of functional outcomes but reported higher numbers of complications, predominantly in the orthosis group, compared to this review [13, 38–40]. These differences in complication numbers are caused by the dominance of surgical wound-related complications (e.g. wound dehiscence or infection) in the post-surgical groups [37]. This high attribution of soft-tissue complications could be caused by friction between the NOM and the surgical site, resulting in higher numbers of superficial wound infections of the surgical wound [41]. No statistically significant differences were found regarding types of complications (e.g. DVT, joint stiffness, or complex regional pain syndrome) [35, 38, 39].

Functional outcome was good to excellent in both NOM-groups and is partly in line with previous study results [13, 38–40, 42]. This study describes no statistically significant differences at 6 or 12 weeks, but does describe statistically significant differences at or after 26 weeks in favour of the removable orthosis. The 95% CI of the 6 weeks and 26 weeks result did not overlap the MCID of 4.4 points, resulting in non-inferiority in terms of functional outcome for a removable orthosis compared to a cast at these two time points. Previous studies have described similar or significantly improved functional outcomes in favour of the removable orthosis compared to a cast in the short-term (less than 12–16 weeks) and no significant differences in both groups in the long-term (over 16 weeks) [13, 38, 39]. Even though these studies included multiple types of ankle fractures, of which some required surgery, functional outcome was non-inferior in a removable orthosis compared to a cast for short-term results and not statistically significant for long-term results.

### Factors that influence recovery

Three factors that seem to influence recovery in NOM are the rigidity of the used management, the permission of weight-bearing, and the duration of NOM.

The rigidity of removable orthoses in the included studies varied from rigid (e.g. long walking boots) to flexible, minimally supporting variants (e.g. elastic support or bandages), all resulting in good functional outcome. These functional scores are in line with a study comparing two types of orthosis in stable type B ankle fractures [43]. The fundamental similarity in the used orthoses is that they restrict the repetition of supination and external rotation (the trauma mechanism of the treated ankle fracture). These findings suggest that even minimal support is sufficient in the NOM of stable type B ankle fractures. All patients were allowed full weight-bearing directly after the fracture was deemed stable. In three studies, the duration of NOM was shortened to 3 or 4 weeks [31–33]. This resulted in secondary displacement, delayed union, or non-union in only eight out of 303 (%) patients in the (rigid) cast group in three studies [30–32].

Since complications were low and functional outcomes were excellent, NOM that restricts repetition of the trauma mechanism, early weight-bearing, and shortened duration of NOM seem a justified option for stable type B ankle fractures.

### Strengths and limitations

To our knowledge, this is the first literature overview that compares NOM for stable type B ankle fractures. The meta-analyses give profound insight into two important outcomes for NOM: complications, describing the safety of NOM with an orthosis or cast, and functional outcome. The comprehensive search strategy led to a minimal chance of missing relevant studies and gave a realistic overview of all published information. Using a small scope of included injuries in the search strategy made this review and meta-analysis more applicable to daily practice. This is in line with a recent appraisal of a published study. This study included a broader range of ankle fractures prior to immobilization [38, 44]. Furthermore, this literature review could contribute to the decrease in secondary healthcare utilization, healthcare costs and societal costs related to these injuries. If fractures are deemed stable and nonoperative management does not require substitution (e.g. change of cast), routine physical outpatient follow-up is debatable [42, 45, 46]. Previous studies showed that stable type B fractures could successfully be treated without additional face-to-face follow-up or imaging [42, 47, 48].

This study has several limitations that need to be addressed. First, selection bias may have been introduced because of the heterogeneity in the used classification systems, diagnostic methods and criteria to exclude additional medial or posterior injury. This may have led to an incorrect selection of patients with a ‘true’ Lauge-Hansen ankle fracture. Recently two reviews aimed to pinpoint one optimal strategy to exclude medial or posterior injury in stable type B ankle fractures, but no consensus has been reached [10, 49]. Second, the small number of studies, with relatively small sample sizes, conducted on this issue reduces the generalizability of findings. Last, the length of follow-up and timing of OMAS varied across studies and affected functional outcome as they were measured at different stages of healing. To minimalize this limitation, all corresponding authors were contacted for missing data, and data were converted to comparable estimations.

### Clinical implications and future perspectives

Current literature describes promising results in terms of complications and functional outcome for a removable orthosis compared to a cast as NOM for stable type B ankle fractures. After stability assessment, using a removable orthosis for patients with a stable type B ankle fracture is advisable. No consensus has been reached on optimal techniques to assess stability. Future research should focus on developing a practical, more sensitive and specific technique to exclude additional medial or posterior injury. To increase the comparability of future studies, a more uniform design in terms of classification systems, endpoints of follow-up, and short-term and long-term outcomes to evaluate ankle fractures should be used. Furthermore, limited evidence on the optimal rigidity of removable orthosis exists, and should be further investigated to optimize NOM after stable type B ankle fractures.

## Conclusion

Results of this systematic review and meta-analysis show that a removable orthosis is a safe alternative type of NOM, as complication numbers are significantly lower in the orthosis group. In addition, the 6-week and the 26-week OMAS results show that in patients with stable type B ankle fractures, a removable orthosis is non-inferior to a cast in terms of functional outcome.

## What is already known on this topic?

- Traditionally, stable type B ankle fractures were managed with a cast, providing maximum bone support during healing.
- Cast immobilization can result in joint stiffness, muscle wasting and DVT; removable orthoses might help with these problems by allowing earlier mobilization and permissive weight-bearing.
- Stable type B fractures can adequately be treated with NOM.

## What this study adds?

- Removable orthosis result in fewer complications than a cast after a stable type B ankle fracture.
- No statistically significant differences regarding short-term functional outcome were found between a removable orthosis and a cast in patients with a stable type B ankle fracture.
- A removable orthosis is non-inferior compared to a cast as NOM regarding functional outcomes at 6 weeks and at 26 weeks or after in patients with a stable type B ankle fracture.

## Appendix 1

Search syntax used in PubMed, Embase, and Cochrane to identify eligible studies for a systematic review of type B ankle fractures comparing removable orthosis and a cast as nonoperative management

Pubmed: 15th July 2022

("ankle fractures"[mesh] OR "ankle injuries"[mesh]) OR (("weber B"[tiab] OR “type B”[tiab] OR "Lateral malleolus"[tiab] OR “distal fibula” [tiab] OR "fibula*"[tiab] OR “Supination-eversion”[Tiab] OR “Supination-exorotation” [tiab] OR “SE-II”[Tiab] OR “Lauge Hansen”[Tiab] OR “Lauge-Hansen”[tiab] AND "fracture*"[tiab])) AND ("braces"[MeSH] OR "brace*"[tiab] OR "bracing"[tiab] OR "Orthotic Devices"[Mesh:NoExp] OR "Orthotic Device*"[tiab] OR "Foot Orthoses"[Mesh] OR "orthoses"[tiab] OR "orthosis"[tiab] OR "non-operative*"[tiab] OR "nonoperative*"[tiab] OR “conservative*” [tiab])

Embase: 15th July 2022

('ankle fracture'/exp OR 'ankle fracture' OR 'ankle injury'/exp OR 'ankle injury' OR (('weber b':ti,ab OR 'type b':ti,ab OR 'lateral malleolus':ti,ab OR 'distal fibula':ti,ab OR 'fibula*':ti,ab OR 'supination-eversion':ti,ab OR ‘supination-exorotation’:ti,ab OR 'se-ii':ti,ab OR 'lauge hansen':ti,ab OR 'lauge-hansen':ti,ab) AND 'fracture*':ti,ab)) AND ('brace'/exp OR 'brace' OR 'brace*':ti,ab OR 'bracing':ti,ab OR 'orthosis'/exp OR 'orthosis' OR 'orthotic device*':ti,ab OR 'foot orthosis'/exp OR 'foot orthosis' OR 'orthoses':ti,ab OR 'orthosis':ti,ab OR 'non-operative*':ti,ab OR 'non operative*':ti,ab OR 'conservative*':ti,ab)

Cochrane: 15th July 2022

(weber B:ti,ab OR type B:ti,ab OR Lateral malleolus:ti,ab OR distal fibula:ti,ab OR fibula*:ti,ab OR Supination-eversion:ti,ab OR supination-exorotation:ti,ab OR SE-II:ti,ab OR Lauge Hansen:ti,ab OR Lauge-Hansen:ti,ab) AND fracture*:ti,ab AND (brace*:ti,ab OR bracing:ti,ab OR (Orthotic NEXT Device*):ti,ab OR orthoses:ti,ab OR orthosis:ti,ab OR nonoperative*:ti,ab OR nonoperative*:ti,ab OR conservative*:ti,ab)
